# A Survey of Active Learning for Quantifying Vegetation Traits from Terrestrial Earth Observation Data

**DOI:** 10.3390/rs13020287

**Published:** 2021-01-15

**Authors:** Katja Berger, Juan Pablo Rivera Caicedo, Luca Martino, Matthias Wocher, Tobias Hank, Jochem Verrelst

**Affiliations:** 1 Department of Geography, Ludwig-Maximilians-Universität München (LMU), Luisenstr. 37, 80333 Munich, Germany; 2 Secretary of Research and Graduate Studies, CONACYT-UAN, 63155 Tepic, Nayarit, Mexico; 3 Department of Signal Processing, Universidad Rey Juan Carlos (URJC), Mostoles, 28933 Madrid, Spain; 4 Image Processing Laboratory (IPL), Parc Científic, Universitat de València, Paterna, 46980 València, Spain

**Keywords:** Gaussian process regression, EnMAP, hyperspectral, query strategies, optimal experimental design

## Abstract

The current exponential increase of spatiotemporally explicit data streams from satellitebased Earth observation missions offers promising opportunities for global vegetation monitoring. Intelligent sampling through active learning (AL) heuristics provides a pathway for fast inference of essential vegetation variables by means of hybrid retrieval approaches, i.e., machine learning regression algorithms trained by radiative transfer model (RTM) simulations. In this study we summarize AL theory and perform a brief systematic literature survey about AL heuristics used in the context of Earth observation regression problems over terrestrial targets. Across all relevant studies it appeared that: (i) retrieval accuracy of AL-optimized training data sets outperformed models trained over large randomly sampled data sets, and (ii) Euclidean distance-based (EBD) diversity method tends to be the most efficient AL technique in terms of accuracy and computational demand. Additionally, a case study is presented based on experimental data employing both uncertainty and diversity AL criteria. Hereby, a a simulated training data base by the PROSAIL-PRO canopy RTM is used to demonstrate the benefit of AL techniques for the estimation of total leaf carotenoid content (*C_xc_*) and leaf water content (*C_w_*). Gaussian process regression (GPR) was incorporated to minimize and optimize the training data set with AL. Training the GPR algorithm on optimally AL-based sampled data sets led to improved variable retrievals compared to training on full data pools, which is further demonstrated on a mapping example. From these findings we can recommend the use of AL-based sub-sampling procedures to select the most informative samples out of large training data pools. This will not only optimize regression accuracy due to exclusion of redundant information, but also speed up processing time and reduce final model size of kernel-based machine learning regression algorithms, such as GPR. With this study we want to encourage further testing and implementation of AL sampling methods for hybrid retrieval workflows. AL can contribute to the solution of regression problems within the framework of operational vegetation monitoring using satellite imaging spectroscopy data, and may strongly facilitate data processing for cloud-computing platforms.

## 1 Introduction

In view of the unprecedented data availability delivered by recently launched and planned optical satellite missions, agricultural and other ecosystem applications will benefit largely from the provided up-to-date information regarding vegetation status and dynamics [1]. For these purposes, the remotely sensed signals must be translated into essential vegetation variables or functional traits at both leaf and canopy levels. Traits can be of morphological, biochemical, physiological, structural or phenological nature [2], for instance leaf area index (LAI), leaf water or pigment contents. With the launch of the Copernicus mission Sentinel-2 in 2015 (and 2017) as well as in view of upcoming satellite imaging spectroscopy missions, such as the Environmental Mapping and Analysis Program (EnMAP [3]) and the high-priority mission candidate Copernicus Hyperspectral Imaging Mission for the Environment (CHIME, [4]), big data streams are going to increase. Hence, efficient and accurate methods for mapping and monitoring of vegetation properties from these Earth observation (EO) data are required. In the last five decades, numerous retrieval methods have been proposed and developed to predict biophysical and biochemical vegetation traits from EO data, ranging from parametric and nonparametric regressions to physically-based and hybrid approaches [5–7]. Since these studies provide exhaustive and up-to-date taxonomies of quantitative retrieval methods, we will concentrate here on the recently promoted hybrid retrieval workflows [8–12]. Hybrid retrieval strategies denominate a combination of radiative transfer models (RTM), providing physical constraints and domain knowledge [13], with fast and flexible machine learning (ML) regression algorithms. In such a framework, typically conventional single (shallow) models are being used, whereas the development of deep learning (DL) models has not yet been applied frequently [14]. Two widely used RTMs in vegetation modelling studies are the leaf optical properties model PROSPECT (recent version PROSPECT-PRO [15]) and the Scattering by Arbitrarily Inclined Leaves (SAIL) [16,17]. The models are usually coupled to simulate canopy bidirectional reflectance from 400 to 2500 nm as a function of several biochemicals, such as pigment, protein and water contents, and biophysical input parameters, such as LAI, average leaf inclination angle, spectral soil background, as well as observation and viewing geometries [18,19]. This modelling scheme, here called PROSAIL-PRO, can be used to establish training databases composed of vegetation properties (=RTM input) and simulated spectral signals (=RTM output), also known as look-up-tables (LUT). In the study by Weiss et al. [20], LUTs were introduced as robust physically-based inversion methods in the context of vegetation properties retrieval from remote sensing data using PROSAIL. Extending this idea to the framework of a hybrid approach, the selected ML regression algorithm is run over such a predefined LUT, or training database, to learn the inherent patterns and nonlinear relations between input and output. Finally, the established retrieval models can be applied over full satellite scenes to map vegetation functional traits in a fast and efficient manner. Recently, kernel-based algorithms have been successfully exploited for such a retrieval scheme, namely Gaussian process regression (GPR) [21,22]. GPR is based on a probabilistic treatment of regression problems which leads to an analytical expression of the predictive uncertainty, provided along with final estimates of functional traits [14,23]. Together with the high accuracy achieved with these algorithms, this specific characteristic renders GPR particularly attractive for solving EO regression problems: information of uncertainty in the model parameterization or input data [24] can be used to assess the models transferability to other locations and times [25]. Many alternative ML approaches have been proposed and applied typically based on artificial neural networks or random forest regression [6,12,26,27]. However, these algorithms do not have the evident advantage of delivering associated uncertainty, which makes them less attractive for vegetation mapping applications.

In the context of PROSAIL model inversion based on the LUT-strategy, the proposed number of samples ranged up to 100,000 [20,28–30] combinations of input parameters. With this size, the training datasets were considered as representative for a wide range of environmental situations. However, this point of view may also be traced to the radiometric approach of physically-based inversion: the solution here is calculated by means of a cost function that minimizes between simulated and measured spectral signatures. Since LUT-based inversion strategies only exploit radiometric information, usually the larger the table, the more accurate was the retrieval. Though, this also depends on the applied strategy, for instance the fraction or number of simulations selected for calculation of the solution [20,30].

When moving towards hybrid approaches using kernel-based machine learning algorithms, processing becomes computationally unfeasible with these large training datasets. Moreover, retrieval results may be biased through unrealistic and redundant parameter combinations within the data pool. This implies that a balance is needed: the characteristics of the training dataset should be a trade-off between realistic sampling and lowest possible size. Dimensionality reduction (DR) is key to tackling the problem of data size. DR can be done in the two dimensions of the training dataset, i.e., in the (1) spectral domain, referring to the number of bands, and (2) in the sampling domain, which specifies the number of available samples or the size of a training dataset [31]. In respect of (1), feature engineering and feature extraction methods offer the opportunity to condense data space, and at the same time remove noise and redundant data [31,32]. These methods are particularly relevant when using hyperspectral data, where multicollinearity leads to suboptimal regression models. However, for hybrid retrieval approaches reduction in the spectral domain may not be sufficient regarding the huge number of possible samples for instance generated by RTMs, and therefore also reduction in the sampling domain (2) is required. A solution to the sampling reduction problem is given by semi-supervised approaches, in which unlabeled samples are exploited during the design of the regression model [33]. These techniques are also known as active learning (AL), aiming to optimize training datasets through intelligent sampling by means of an iterative procedure [34].

With this background in mind, our study was narrowed down to the following objectives:

- Provide an up-to-date overview of the use of AL heuristics in the framework of biophysical and biochemical vegetation traits retrieval from terrestrial EO data;
- Identify optimal AL strategies to obtain efficient training datasets for kernel-based ML regression algorithms to be implemented in hybrid retrieval workflows;
- Give recommendations and inspirations for further research under the AL perspective and in the context of terrestrial vegetation monitoring from EO data.

To achieve this, a thorough summary of AL and critical analysis of the available literature is carried out in the context of estimating functional vegetation traits from EO data over terrestrial surfaces. We further present two examples employing different AL techniques to demonstrate the efficiency of this approach. Finally, challenges and future perspectives for implementing AL heuristics in retrieval workflows are summarized.

## 2 Background: Active Learning Theory

Generally, sampling reduction or “scaling down” techniques can be categorized into three types: random sampling (RS) [35], active learning (AL) [36,37] and progressive sampling (PS) [38]. Random sampling techniques are the simplest approaches to reduce a dataset. However, in RS neither attempts are made to render the final training dataset as informative as the entire data pool, nor the smallest possible sample is sought for [39]. Progressive sampling methods make use of the concept of the learning curve [38]. Hereby, the algorithm learns on an initial sample, and then gradually increases this sample until the learner’s accuracy no longer improves. PS sampling attempts to increment training samples only up to the point at which model accuracy reaches a plateau. There are some issues with PS that have to be considered. For instance, if samples are grown too rapidly, the training dataset can become “overshot”, meaning that it will be larger than required. In contrast, if the sample is increased in only small increments, the computational demand for convergence testing may be too high [39]. Hence, among these three categories, AL is an auspicious technique recently applied within many machine learning problems where labeling of data is difficult, time-consuming or expensive [36]. In statistics, this is also known as “query learning” or “optimal experimental design”. The key idea behind AL is that a ML algorithm can obtain higher accuracy with fewer training data if it is allowed to choose the data from which it learns [36]. According to Settles [36], who provided the first large-scale survey of AL literature, three different problem scenarios in which the learner is able to ask queries can be identified: (1) membership query synthesis, (2) streambased selective sampling, and (3) pool-based sampling. In the context of EO analysis and modelling, AL has mainly been used in three applications:

1. Classification, e.g., [34];
2. Emulation, e.g., [40];
3. Regression, e.g., [41].

Within all three fields, the main task is to generate a sample of fewer data, which often confronts two competing requirements [39]:

- The sample must be (nearly) as informative as the full dataset, implying that a learning algorithm can extract the same essential information from the sample as it would from the full dataset [35];
- The sample should be as small as possible in order to reduce the computational load.

Both requirements can be optimally addressed by AL techniques, which present an intelligent sampling step selecting the most informative samples from a large training data pool. In this way, redundancy is avoided, which usually leads to decreased accuracy; and at the same time the training dataset is effectively reduced to allow fast computing. Hence, AL heuristics have a great potential to optimally design training samples that can be generated by RTMs [27,41].

### 2.1 Active Learning for Classification

When inspecting the existing literature on the subject of AL in remote sensing, it appears that these methods have mainly been used for classification problems [34,42–44]. For instance, in the study by Tuia et al. [34], state of the art approaches of AL for classification of remote sensing images are presented and compared. A series of heuristics were classified into four families, which are: (1) committee-based heuristics, (2) large margin-based heuristics, (3) posterior probability-based heuristics, and (4) cluster-based approaches. According to Kumar and Gupta [45] query strategies for classification can be further divided into: informative-based, representative-based, and informative- and representative-based approaches. This study also critically discusses some recent efforts to combine reinforcement learning and DL with AL [45,46].

For classification problems, usually human experts are needed to assign labels to the data. This process, however, is often costly and requires solutions to reduce these efforts [47]. AL has hereby emerged as the most popular approach to build classification models from human supervision. In this respect, enormous progress has been made on instance-based query strategies. Moreover, several groups of AL solutions have been proposed, e.g., [48]. Further approaches include hierarchical cluster to order and select instances [49], or batch-mode active learning (BMAL) [50]. The interested reader is referred to some representative classification studies using and reviewing AL strategies [34,45,51–53].

Although not particularly treated in this study, clustering-based measures of representativeness should be mentioned [54]. Hereby, AL can be used to query data instances to be merged with conventional clustering algorithms for improving clustering quality [45].

### 2.2 Active Learning for Emulation

As an interesting application of AL-based sampling optimization, new AL techniques have recently been implemented to construct surrogates of complex deterministic models. This statistical technique of approximating the functioning of a physical model is termed “emulation”, and has been demonstrated for a number of canopy and atmosphere RTMs [55–58]. In emulation, efficient machine learning algorithms are being constructed to replace the input-output functioning of computationally costly complex models. The emulator is derived (trained) from a relatively small number of model runs covering a multidimensional input space. Once the emulator is built, it is not necessary to perform any additional run with the model, regardless of how many analyses are required to assess the simulator’s behaviour. Hence, a key aspect is to identify the optimal number of simulations in order to build an emulator with maximal accuracy. In this respect, AL frameworks are being developed that sequentially choose informative input points [14,40]. The latter methodology is mainly based on the notion of an acquisition function (AF), which can be optimized through gradient-based techniques, similar to Bayesian Optimization.

The related literature is wide. Several research areas have addressed the same problem (i.e., AL for regression/emulation) under different names, such as optimal experimental design [59–62], optimal sensor placements [63,64], generation of quasi-random uniform sequences (Latin Hypercube sampling), Sobol sequences [65,66] and determinantal point processes [67,68], non-uniform, adaptive sampling and quantization of a signal [69,70]. Moreover, adaptive quadrature rules [71,72] and approximations of posterior densities have been introduced [73,74]. Hereby, the notation of AF, explicitly or implicitly defined, is the key point of all these techniques. Different criteria have been used for designing suitable AFs based on:

- The maximization of the predictive variance of the emulation/regression model [62];
- Space filling procedures [66–68];
- Combinations of the previous two strategies (maximization of the predictive variance and space filling) [14,40,71,72];
- The minimization of the prediction error using a Cross-Validation (CV) procedure [75,76];
- The maximization of the entropy or the mutual information [63,64].

Further, AFs can be based on classical statistical criteria. Mathematically speaking, the traditional optimal criteria for the experimental design are functions of the eigenvalues of the Fisher information matrix related to the model to emulate [59,60]. The most famous criteria of this class are [59–61]:

- A-optimality;
- D-optimality;
- E-optimality.

A-optimality seeks to minimize the trace of the inverse of the Fisher information matrix. In linear regression, this criterion results in minimizing the average variance of the estimates of the regression coefficients. D-optimality seeks maximizing the determinant of the Fisher information matrix, and E-optimality maximizes the minimum eigenvalue of the Fisher information matrix [59–61].

### 2.3 Active Learning for Regression

Moving towards regression applications, progress in solving problems with AL seems comparatively lower than in the fields of classification or clustering [45]. Whereas in classification applications the samples are labelled by a human expert (oracle), in the case of AL for regression solutions and remote sensing, usually a large pool of unlabeled samples is gathered at once, for instance by simulating a training dataset using RTMs or by field data collections. Hence, the category of pool-based sampling [77] is of most interest, and the need for human experts becomes obsolete. Traditionally, ML regression algorithms work rather passively by receiving labelled data information. The major obstacle to obtain successful retrievals comes from the machine’s inability to distinguish between low-level and high-level semantic meanings of these training samples [78]. AL overcomes this bottleneck by enabling the learner (machine) to collect data according to defined selection criteria. Hence, a statistically “optimal way” to select the most meaningful training samples can be performed by the machine itself.

Two families or query frameworks were adapted to solve regression problems for Earth observation data analysis [33,41]: (1) uncertainty, e.g., [79] and (2) diversity, e.g., [80].

#### 2.3.1 Uncertainty Criteria Methods

Uncertainty sampling heuristics are perhaps the most simple and most frequently applied query frameworks. Hereby, samples were ranked according to their uncertainty: subsequently, only those samples were selected by the algorithm that have the least certainty [39]. Here we can further distinguish between variance-based pool of regressors (PAL), entropy query-by-bagging (EQB), or residual regression active learning (RSAL) [34,81,82].

PAL, for instance, at first generates *k* subsets by randomly choosing samples (*y_i_*) from the original training set. Each subset is then used to train a regressor and to obtain a prediction for each sample in the candidate set. Finally, *k* different predictions for each candidate sample are obtained. Then, the variance of each prediction ($\sigma_{y}^{2}$) is estimated as:

(1) $$\sigma_{y}^{2}=\frac{1}{k}\overset{k}{\underset{i=1}{\sum^{+}}}{\left(y_{i}-\bar{y}\right)}^{2},$$

where $\bar{y}=\frac{1}{k}\sum_{i=1}^{k}y_{i}$. The variance gives an indication of the spread of the estimations. Samples with highest variance are added to the training set. The other two heuristics (EQB, RSAL) are explained in detail in Appendix A.1 (Uncertainty criteria methods) and in Verrelst et al. [41].

#### 2.3.2 Diversity Criteria Methods

Choosing samples according to their diversity means that added samples are dissimilar from those already implemented in the training dataset. Here we distinguish between Euclidean distance-based diversity (EBD), angle-based diversity (ABD), and cluster-based diversity (CBD) [81–83].

As an example, the EBD method [81] selects those samples out of the pool that are distant from the already included ones in the training set, using squared Euclidean distance:

(2) $$d_{E}={\left\|x_{u}-x_{l}\right\|}_{2}^{2},$$

where *x_u_* is a sample from the candidate set, and *x_l_* is a sample from the training set. All distances between samples are computed and then the farthest are selected. The other two heuristics (ABD, CBD) are explained in detail in Appendix A.2 (Diversity criteria methods) and in verrelst et al. [41].

## 3 Literature Survey: Active Learning under the Earth Observation Perspective

A systematic literature analysis was carried out using predefined criteria. Unlike traditional review studies, the purpose of such a systematic review is to provide a complete list of all published studies using a rigorous and well-defined approach to identify relevant literature in a specific subject area [84].

### 3.1 Systematic Approach

Since our objective is focused on the AL perspective for solving Earth observation regression problems, we searched for articles in Web of Science using the keywords “active learning” and “regression” and “remote sensing”, resulting in a total of 16 records (access date: 16 October 2020). Conference proceedings and technical reports were excluded from the results. Further, we searched in these records for other potentially relevant studies. Finally, six peer-reviewed articles fulfilled our pre-defined criteria: AL techniques in the context of estimating functional vegetation traits from EO data over terrestrial targets. Table 1 gives an overview of these studies, summarizing sensors used, estimated vegetation traits, implemented ML algorithms and applied AL methods. The first study was published in 2016 [41], and four were from 2020 suggesting increasing interest in these rarely used techniques.

Three studies [33,85,86] are not listed in Table 1 since their experiments were not focused on terrestrial surface variables. Yet, they introduced AL in the context of remote sensing and regression solutions for biochemical variable retrieval: at first, AL methods were used for the inversion of radiative transfer calculations aiming to estimate chlorophyll a, coloured dissolved organic matter and suspended particulate matter in the Caspian Sea using MEdium Resolution Imaging Spectrometer (MERIS) data [85]. Pasolli et al. [33] demonstrated the efficiency of AL on Sea-viewing Wide Field-of-view Sensor (SeaWiFS) data to estimate chlorophyll concentration in coastal and open waters. The study by Douak et al. [86] introduced AL for the estimation of chemical concentration from spectroscopic data.

Moreover, a recent study proposed an active learning regularization (ALR) approach to increase the clear sky retrieval rate. Briefly, a local, variable specific, representative calibration database was generated, which was further used to select a subset of informative vegetation indices to establish local regression predictor [87].

Important to mention in this context are some first attempts to optimize a training database by Baret et al. [88]. The authors already proposed an optimal design of the training data pools (for a neural network algorithm), though not actually using AL. In that study, the training database was streamlined in the reflectance space, retaining those cases belonging both to the simulated and actual remote sensing measurements. Here, a threshold based on the minimum root mean square error (RMSE) value was defined, used to decide whether a simulated case is rejected or included in the training database. Further, application of additional criteria to efficiently streamline a training dataset were proposed [88,89].

### 3.2 Sensors and Estimated Variables

At first, AL heuristics were investigated on simulated Sentinel-3 OLCI data, evaluating the theoretical performance of the resulting models trained on simulated resampled PROSAIL datasets [41]. With exception of the study by Verrelst et al. [90], who used proximal sensing spectroscopy to simulate EnMAP sensor data, mainly multispectral data sources were exploited, such as Landsat-8 OLI [92], or superspectral, such as Sentinel-2 [27,93] or VEN*μ*S [91] (see also Table 1).

Diverse vegetation functional traits were estimated by the selected studies, focusing mainly on LAI [27,41] (or green LAI [93]) and leaf chlorophyll content (*C_ab_* [27,41,92]), but also fractional vegetation coverage (Fcover [27,91]), fraction of absorbed photosynthetically active radiation (fAPAR [27]) and aboveground nitrogen content (*N_area_* [90]). Results of the selected studies suggest that AL methods were sensitive to the studied variables. For instance, Verrelst et al. [41] found that *C_ab_* retrieval with kernel ridge regression (KRR) led to similar results when applying diversity AL methods EBD, ABD, and CBD, along with the uncertainty methods PAL and EQB. In the case of LAI, however, all coefficient of determination (R^2^) values were generally lower. Though, optimal results for LAI converged faster, with maximum of 400 samples compared to 500 for *C_ab_*. This may also be due to the different ML methods used (GPR versus KRR, see also Section 3.3). Nonetheless, also the study by Upreti et al. [27] identified different patterns of convergence depending on the variable of interest. In their study, the authors tested the estimation of LAI, *C_ab_*, fAPAR, Fcover, and canopy chlorophyll content from Sentinel-2 data using different algorithms [27].

### 3.3 Applied ML Algorithms and AL Heuristics

For the purpose of exploring AL strategies specifically for regression models, kernelbased methods were exclusively implemented in the selected studies (Table 1). For instance, two of these algorithms were exploited by Verrelst et al. [90] for joint usage within the retrieval workflow. Fast KRR [94], presenting an ideal method to carry out time consuming simulations [5], was used at first to minimize and optimize the training data base using six different AL heuristics. In a next step, variational heteroscedastic Gaussian process regression (VHGPR) was employed to accurately map *N_area_*. VHGPR models have been shown to outperform standard GPR models in terms of accuracy and provided more realistic uncertainty information [8,10], though at the cost of a slightly longer training time. The combination of these two algorithms led to optimization of the retrieval workflow: KRR is the faster method, but VHGPR provides uncertainties of the estimates. As a consequence of their fast and reliable performance, the majority of studies used standard GPR approaches, which often ranked as the top algorithm in theoretical performance and when validating against in situ reference data [27,91–93].

Regarding implemented AL methods, mainly diversity and uncertainty selection strategies have been successfully tested by the selected studies. Verrelst et al. [41] concluded that EBD, ABD and CBD performed fastest and (partly) obtained highest retrieval accuracy over tested variables. Random sampling, in contrast, did not offer a stable error minimization. Based on these findings, Upreti et al. [27] applied these three diversity criteria for the optimization of a simulated training dataset based on PROSAIL to estimate diverse biochemical and biophysical variables from Sentinel-2 data. The authors concluded that the performance of the EBD methods surpassed those of ABD and CBD strategies [27]. Further, they found that a full training dataset of 2500 samples provided lower retrieval accuracy than a smaller dataset optimized with AL heuristics. This was explained by the fact that a largely sampled training dataset may include redundancy leading to decreasing retrieval performance. In a follow-up study [91], EBD was directly applied within the retrieval workflow for the estimation of Fcover from VEN*μ*S satellite data. Retrieval accuracy was good with R^2^ of 0.76 and relative RMSE (rRMSE) of 11.6%, though a slight overestimation of high Fcover values and underestimation of low values was observed. Zhou et al. [92] compared the six AL methods proposed in [41], and found that EQB was best performing for the retrieval of *C_ab_* from Landsat-8 OLI data. However, EBD provided highest accuracy from all diversity methods, and best theoretical performance together with CBD. Moreover, the study [92] confirmed that significant lower retrieval accuracy was obtained when the full dataset was used (rRMSE of 56%) compared to optimized AL sampling using EQB or EBD (rRMSE of 22% and 25%, respectively). Likewise, the study by Verrelst et al. [90] obtained optimal results when using the EBD method for the estimation of *N_area_*. Second best results were achieved by PAL, however, this method required 4.7 times longer computational time for sample selection than EBD. This aspect of computational load for the learning process has to be considered, in particular when kernel-based methods are employed. In a GPR training process, the inverse of the co-variance matrix is computed and the time required to evaluate it scales cubically [95]. The study from Pipia et al. [93] also obtained optimal results with EBD in the context of green LAI retrieval from Sentinel-2 data. Specifically, in this study, an adaptation of the standard GPR formulation was proposed to allow parallel processing and integration into Google Earth Engine (GEE [96]). Hereby, again the six different AL methods were tested, i.e., ABD, CBC, EBD, EQB, PAL and RSAL. Results indicated that EBD proved the most capable algorithm to provide a green LAI GPR model both in accuracy and time efficiency. Therefore, EBD-based AL methods were implemented in the retrieval workflow and finally into the GEE environment [93].

The sizes of the used training datasets may seem rather small compared to the typical sample sizes used within radiometric LUT-based inversions strategies, or applied for training of neural networks [20,97]. Note that a standard implementation of a GPR typically can not cope with more than 5000 samples within reasonable time. However, this apparent limitation is well compensated by the algorithms, which require only relatively small training datasets, can adopt very flexible kernel functions and also identify the relevant bands and observations for establishing nonlinear relationships between spectral observation and variables of interest [98]. Four of the studies selected by our review [27,41,91,92] used 2500 samples as a full dataset. Applying AL heuristics led to a reduction of the training dataset to 400−500 samples for LAI and *C_ab_* [41] or 1200−1800 samples for multiple traits [27]. Pipia et al. [93] and Verrelst et al. [90] used only 1000 samples as full training dataset, resulting in optimal size of 218 samples for green LAI retrieval [93]), and 191 for *N_area_* estimation when running against an experimental validation dataset [90], respectively. In all cases, AL-reduced datasets significantly outperformed the accuracy obtained when using the full datasets. The final number of samples not only depends on the original size of a training data pool, but it also depends on the targeted variable, available validation data and applied ML algorithm. Int conclusion, the quality of the training data tends to be more important than the quantity.

## 4 Experimental Case Study

Following the promising results obtained by the selected studies, we demonstrate the efficiency of AL heuristics for the estimation of two important biochemical crop traits. For this purpose, the retrieval of leaf water content (*C_w_*), in cm, and total leaf carotenoid content (*C_xc_*), in μg/cm^2^, is presented from a hyperspectral experimental dataset. *C_w_* is defined as the area-weighted leaf moisture content and is related to a range of physiological aspects. In particular it can be used for evaluating vegetation physiological status, for instance when drought events occur leading to changes in most vegetation types [99]. Further, the detection of water stress is important for agricultural management and can be supported by mapping this essential biochemical variable over time and space. Leaf carotenoids are pigments of major importance for crops achieving two main complementary and indispensable functions in the photosynthetic pathway of higher plants: light harvesting and photo-protection [100]. Along with *C_ab_*, they provide important information about vegetation photosynthetic potential and activity [101].

### 4.1 Data Collection

The two analyzed variables were provided from multiple field trials at a test site in the North of Munich, in Southern Germany: the Munich-North-Isar (MNI) campaigns (N 48°16′ E 11°42′). The MNI site serves for data collection and algorithm validation in the framework of the Environmental Mapping and Analysis Program (EnMAP) [3] for agricultural applications. Data collection was done in the growing periods of 2017 and 2018 over winter wheat (Triticum aestivum) and corn (Zea mays). Measurements included proximal field sensing concurrently (or shortly before) destructive and non-destructive measurements of leaf biochemicals. At the two study sites, a 30 × 30 m grid of nine 10 × 10 m squares was marked out delineating the elementary sampling units (ESU) resembling a future EnMAP pixel. Data were collected at the following dates at the wheat field: 29/3, 10/4, 10/5, 29/5, 13/6, 26/6, 6/7 and 17/7 in 2017 and 12/4, 18/4, 27/4, 7/5, 5/6, 21/6 and 13/7 in 2018. For corn, sampling was done at the following dates: 13/6, 19/6, 26/6, 6/7, 17/7, 18/8, 30/8, 15/9 in 2017 and 15/6, 13/7, 19/7, 26/7, 17/8 and 22/8 in 2018. With this, a total number of 28 measurements was available for validation. Extensive documentation including photographs of selected crop growth stages are provided by Berger et al. [10]. Further, the studies by Danner et al. [102] and Wocher et al. [99] inform about sampling design, size and location of ESUs, as well as measurements of other biochemical and biophysical variables.

To determine in situ *C_xc_*, *C_ab_* was sampled with a specifically calibrated Konica- Minolta SPAD-502 handheld instrument from five leaves at different plant heights and was averaged to receive a representative mean value in μg/cm^2^. Finally, *C_xc_* was derived from *C_ab_* using a linear regression model, which was based on a stable relationship between the two variables for healthy green vegetation [102,103].

For *C_w_* determination, two leaves were randomly cut within each of the defined ESUs (18 samples per date). Leaf samples were weighed, packed in bags and brought to the lab. Final leaf water content in cm was obtained from the mass difference of sample leaves per unit leaf size before and after oven-drying at 105 °C to constant weight.

Hyperspectral signatures of the canopy (within the 350−2500 nm range) were measured along with the biochemicals using the Analytical Spectral Devices Inc. (ASD; Boulder, CO, USA) FieldSpec4 Standard-Res Spectroradiometer. Spectral sampling design consisted of five nadir measurements per ESU at a height of 25 cm above the canopy using a field of view (FOV) of the fiber optic cable of 25°. Throughout the measurements, the sensor was slightly moved over the target while maintaining the nadir angle to collect representative spectral signals capturing the heterogeneity of the canopy. Collected spectra were averaged per ESU and a final mean value was calculated over all nine ESUs to provide a representative reflectance signal of the 30 × 30 m EnMAP-like grid. Processing included splice-correction, white reference baseline calibration, and slight smoothing using a Savitzky-Golay-Filter using frame size of 13 nm [99].

Finally, EnMAP spectral features were simulated from these measurements using spectral full width half maximum (FWHM) value information of the corresponding spectral response functions.

Since the main purpose of optimizing a training dataset is vegetation properties mapping, AL efficiency was demonstrated for spatial retrieval of *C_w_*. Unfortunately, no imaging spectroscopy scene was acquired simultaneously to the in situ data collection at the MNI site. Therefore, we decided to process a well-known HyMap airborne imaging spectroscopy scene, acquired on 12th July 2003 over the Barrax agricultural region, La Mancha in Spain (coordinates 30°3′N, 2°6′W). The flight line is part of the the ESA Spectra Barrax Campaign (SPARC) [104] and has been described and exploited in several earlier studies [10,31,32,105,106]. The Barrax site is characterized by a flat topography and pivot-irrigated fields of alfalfa, corn, potato, winter wheat, sugar beet, garlic and onions, among others. Hence, the same crop types (winter wheat/corn) as at the German test site were present. However, in contrast to MNI, irrigation is required at the Spanish agricultural area, being characterized by semi-arid climate. The HyMap sensor provides 126 spectral bands in the range of 438 nm to 2483 nm with a ground sampling distance of 6 m. Radiometric corrections were performed by the campaigns team according to the procedures described in [107]. For mapping application, FWHM information was used to configure and resample the HyMap scene spectrally to EnMAP. In this way, the GPR models trained on EnMAP spectral characteristics can be applied [10].

### 4.2 Experimental Design

At first, we established a training dataset of 1000 (1k) different combinations from randomly drawing all PROSAIL-PRO input parameters. Leaf input variables of PROSPECT-PRO [15] were sampled as follows: leaf structure parameter: 1.0−2.0, *C_ab_*: 0−80 μg/cm^2^, *C_xc_*: 0−15 μg/cm^2^, *C_w_*: 0.001−0.03 cm, leaf anthocyanin content: 0−2 μg/cm^2^, leaf protein content: 0.001−0.0025 g/cm^2^ and carbon-based constituents: 0.001−0.01 g/cm^2^. Canopylevel input parameters of the 4SAIL model [17] were sampled to: LAI: 0−7 m^2^/m^2^, average leaf inclination angle: 30°−70°, hot spot parameter: 0.01−0.5 m/m, soil brightness factor (scales between one wet and one dry model-implemented soil reflectance): 0−1. The sun zenith angle was set to 30° corresponding to the mean value at all measurements. Abovecanopy reflectance was collected at nadir; hence the sensor observation angle was set to 0°. Parameterization was defined according to previous studies and experience of the authors [10,19,32,108]. The model was then run to simulate corresponding bi-directional vegetation canopy reflectances. Hereby, spectral characteristics were adapted according to the future EnMAP sensor data (242 bands). In this way, the simulated training database and in situ measured samples were spectrally equivalent. Second, principal component analysis (PCA) was applied reducing the simulated spectral features to 20 components. The study by Danner et al. [12], which was also based on simulated EnMAP spectral data, demonstrated that this number is more than sufficient for GPR algorithms to ensure high theoretical estimation accuracy for LAI. Since we demonstrate the AL approach here on two leaf biochemical traits, which are usually more difficult to obtain than canopy variables [25], it was decided to keep this high number of components to only lose minimal information. In a next step, a GPR was used to select most informative spectral samples using the six active learning heuristics, i.e., ABD, CBC, EBD, EQB, PAL and RSAL as well as RS [41,90,92]. Yet, when AL heuristics are employed, we start with an initially annotated dataset (10 samples, or 1%), which was incrementally extended by choosing from the large data pool. Note that the AL algorithm assumes the simulated training database as an unlabeled data pool, and hence iteratively tests a new sample according to a pre-defined query strategy (e.g., EBD or PAL). A new sample is only added when it fulfills the requirement to improve the regression model after being labeled; otherwise, the algorithm proceeds to evaluate the next sample. Optionally, a stopping criterion can be defined, e.g., terminating after 300 samples. Evaluation was done against the in situ sampled field datasets of *C_xc_* and *C_w_* using the RMSE as a statistical measure. Compared to some studies identified by our systematic review [27,41,91,92], the size of the training database is smaller (1000 vs. 2500). However, these studies added 50 samples per iteration. We added only one per iteration to make sure that all samples were evaluated against the validation data.

**Figure 1:**
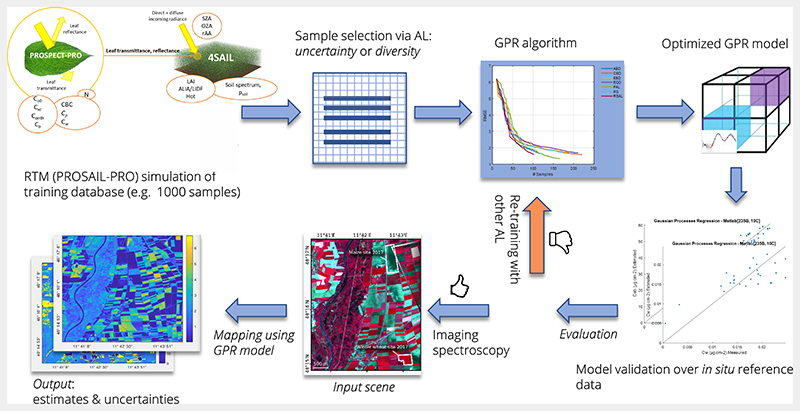
Hybrid retrieval workflow employing PROSAIL-PRO (adapted from [19]). The RTM was used to create the simulated training database, which represents the “unlabeled” data pool. Sample selection is performed with AL heuristics by means of GPR algorithms to establish a specific retrieval model for functional vegetation traits. Output maps provide estimates along with corresponding uncertainty; exemplary maps from Estévez et al. [25].

The corresponding hybrid retrieval workflow including AL-based sample reduction and mapping application is demonstrated in Figure 1.

### 4.3 Evaluation

**Figure 2:**
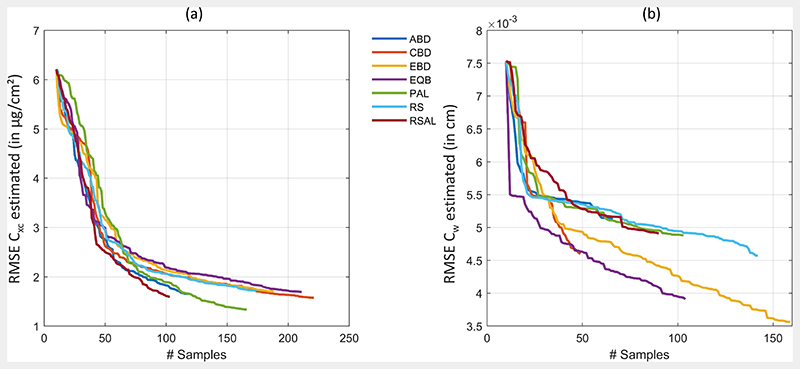
RMSE for retrieval of *C_xc_* (**a**) and *C_w_* (**b**) applying six different AL methods and RS on a PROSAIL-PRO simulated training database with GPR.

Figure 2 demonstrates the efficiency of the AL heuristics. In case of *C_xc_*, optimal results were obtained with uncertainty PAL method, reducing the RMSE from > 6 to 1.33 μg/cm^2^ (rRMSE of 10.2% and R^2^ of 0.88) when trained on 156 samples. No other method showed superior accuracy, but they performed similarly with RMSE < 2 μg/cm^2^ and stopped between 100 and 200 samples, after all samples from the full simulated training database were evaluated. Regarding runtime, EBD proved the most efficient AL method for *C_xc_* estimation, being seven times faster than PAL. In case of *C_w_*, EBD was most convincing, achieving RMSE of 0.0036 cm (rRMSE of 21.2% and R^2^ of 0.77). Second best results were here obtained by EQB method with rRMSE of 23%. Finally, a total number of 150 out of the 1000 samples from the full training dataset was sufficient to provide highest estimation accuracy. EBD was not only obtaining the highest retrieval performance of *C_w_*, it was also the second fastest method closely following CBD.

**Figure 3:**
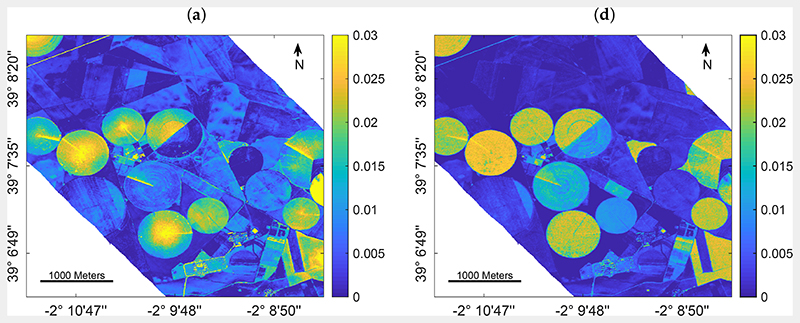

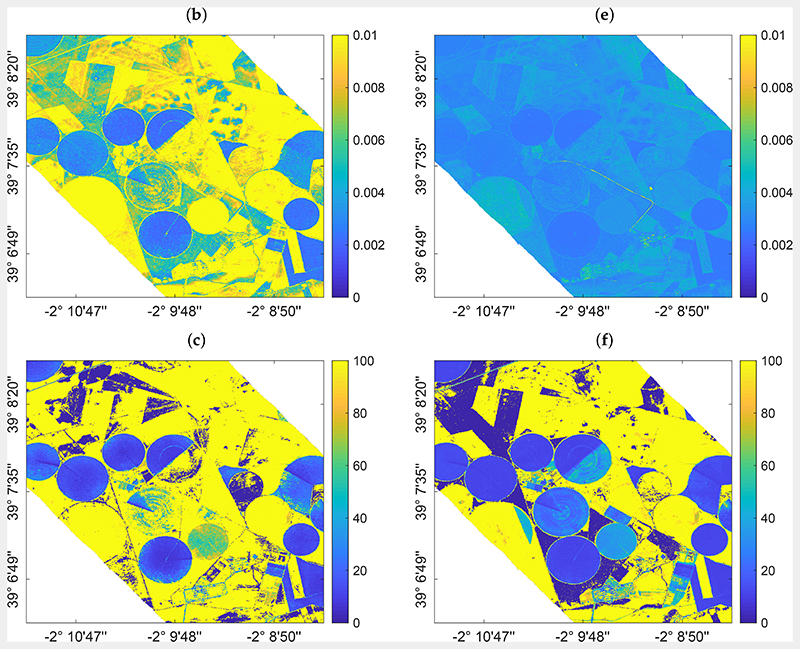
Mapping leaf water content (*C_w_*) using GPR trained over a full training database (left) and using EBD-optimized sampling (right): *C_w_* estimates in cm (**a**,**d**), absolute uncertainty in form of standard deviation cm (SD, **b**,**e**) and relative uncertainty in form of the coefficient of variation % (CV, **c**,**f**).

To demonstrate a spatial retrieval application, GPR models trained over the full dataset (i.e., 1000 samples) and the EBD-optimized training database with only 150 selected samples were compared for *C_w_* mapping using the EnMAP-resampled HyMap scene. To do so, an additional step was required to account for the fact that real images are: (1) more noisy than simulated data, and also (2) consist of non-vegetated surfaces, for which the PROSAIL-PRO model is not optimally configured. Therefore, 5% of Gaussian noise were injected and also 11 soil reflectance samples (including different types of bare soils and crop residues) were added to both training datasets. Figure 3 demonstrates a spatial subset (700 × 700 pixels) of the obtained *C_w_* maps using a full 1k training database (left) versus the EBD-optimized dataset (right) to establish GPR retrieval models.

Since for this scene no in situ reference data was collected for validation, it must be remarked that results can only be interpreted by plausibility. Cropped fields are easily distinguishable through the typical center pivot irrigation systems that characterize the agricultural area. In general, the full training database provided a higher variability of the estimates (Figure 3a) over both vegetated (i.e., crop fields) and non-vegetated areas (i.e., bare fields or fallow land) than the reduced training set (Figure 3d). The higher intra-field variation of estimated leaf water content, and in particular the enhanced *C_w_* in the field centers compared to field borders, appears spatially implausible in view of pivot-irrigated fields: this specific irrigation technique provides equal watering of the crops, hence triggering uniform leaf and plant growth which should be reflected in the retrieval pattern. In contrast to the full simulated training database, retrieval results obtained by the EBD-reduced dataset were more realistic providing equal intra-field distribution of the estimated variable.

This is also in line with the lower uncertainties given by the EBD-reduced simulated training database compared to the full dataset (Figure 3b,e), pointing towards a more realistic mapping approach when AL-based optimization methods are implemented. It is of interest that improvements in uncertainties are especially found over the non-irrigated, dried-out fallow lands. This can be explained by the following mechanisms: First, the reduced training dataset has been optimized against the validation dataset thanks to EBD sampling, thus enabling the trained model to be better adapted for interpreting real data. Second, the added (11) bare soil samples play a more dominant role relative to the reduced training samples as opposed to the full 1k dataset. The more confident estimates using EBD-based selection strategy are also reflected in the relative uncertainty maps (Figure 3c,f). Although the yellow areas indicate high uncertainties, they mark the non-vegetated surfaces. Here, the relative uncertainties appear beyond the given maximum of 100% due to the estimates near to zero going along with uncertainties that are above the near-zero estimates. Nevertheless, when comparing both maps, the EBD-reduced dataset led to a map with substantially more parcels with low uncertainties, thus giving more confidence in the *C_w_* mapping. Altogether, the EBD-reduced training dataset not only led to more realistic estimates, but also provided lower uncertainties as opposed to training with the full simulated data pool.

Two additional remarks are worth noting. First, this experiment only serves for practical demonstration of AL sampling strategies. Further optimization and tests go beyond the scope of the present study, yet similar findings were observed when applied to other experimental hyperspectral datasets (results not shown). Second, AL techniques presented in this study can be tested with the in-house developed software package Automated Radiative Transfer Models Operator (ARTMO) [109]. The AL module was recently updated, e.g., to enable running against validation data, and can be combined with ARTMO’s machine learning regression algorithm (MLRA) toolbox [110]. Eventually, making use of AL sampling strategies may lead to new-generation hybrid retrieval algorithms and opens the door for other applications, as briefly discussed in the following section.

## 5 Towards Advanced Use of Active Learning for Vegetation Properties Retrieval

### 5.1 Discussion of Survey Results

A literature survey was conducted where we systematically identified all relevant papers that investigated AL heuristics using the criteria of solving regression problems within terrestrial Earth observation analysis. Specifically, AL algorithms found their way in hybrid regression strategies when kernel-based ML algorithms are used. One of the main outcomes of this survey is that implementation of AL methods achieved superior retrieval of common biophysical and biochemical vegetation traits compared to the usage of full training datasets (Section 3). The six identified studies also confirmed that tested AL methods led to a smooth convergence to the full training error bound, both for experimental and simulated datasets. This is a highly attractive characteristic of AL-based sample selection, which can be explained by the fact that the strategies only allow the addition of those samples which also improve the overall accuracy of the retrieval model [41]. Obviously, a large training dataset with hundreds or thousands of samples inherently leads to redundancy. Instead, a small selection from the training dataset contains the major information, which can be identified using AL heuristics. Most of the identified studies revealed that EBD heuristics performed superior to the other AL methods. This means that searching for samples that are distant from those already included in the pool proved to be an efficient strategy to converge towards an optimal training dataset. Moreover, testing AL-selected samples directly against in situ collected field data is an efficient method, but compromising generic applicability of the ML model (see discussion in Section 5.2).

To date only AL techniques based on diversity and uncertainty criteria have been implemented in the context of regression for EO data analysis. Since many other query strategy frameworks have been proposed within the classification context (e.g., [34,36,46,50]), these methods may be adapted for regression. For instance, AL based on the density criterion [111] could be implemented in hybrid workflows.

### 5.2 Discussion of Experimental Results

Apart from the literature review, we showcase the mapping capabilities of promising GPR algorithms combined with AL within a hybrid workflow for the retrieval of two essential biochemical traits. Similar to [90], the AL-GPR algorithms were run against an in situ dataset. This has the advantage that the training dataset becomes adaptive against real (noisy) data, and so partly may overcome the common mismatch between simulated and real-world spectra [102,112]. The downside of optimizing against in situ data, however, is running into the risk that the reduced training samples lead to over-specialization to the field study case. In this way it may lose its generic character, which is the essence of hybrid retrieval strategies. To avoid this risk, we can opt to keep the initialization dataset of the AL sequence sufficiently large. This initially annotated dataset could be, for instance, 5% of data from the pool, as shown by [90]. However, the quality of this randomly selected dataset is unknown. Hence, to maximize the impact of the AL method used, we decided to reduce this initial dataset to 1% randomly selected data from the full pool. The process was then iterated until all samples in the training dataset were tested, which assured a lower impact of the initial choice on final results. With a final number of 150 from 1000 samples in the optimized dataset (in the case of *C_w_*), only around 6% of the training data come from the initial set. In this respect, an AL strategy striving for optimal generic applicability and robustness towards real data has yet to be further investigated.

From the experimental results we can conclude that the EBD emerges as the most efficient AL method for solving regression problems in this context: it is one of the methods that delivers the highest levels of accuracy along with the retrieval of multiple functional traits. Moreover, it is the (or one of the) fastest methods in the sample selection process. Regarding *C_w_* mapping speed (Section 4.3, Figure 3), the EBD-reduced training dataset allowed establishment of a model that runs 2.5 times faster than GPR trained over the full training database. Although computational power may not be a limiting factor for these small experiments, when it comes to the acquisition of large spaceborne scenes, lighter models will allow much faster processing. Besides, the size of a final GPR model, which was established using AL-sampling is substantially smaller (5−30%) compared to a model trained over a full training dataset. The reduced model size is another benefit of AL implementation, being essential for storing a final retrieval model within software toolboxes. Furthermore, the provided mapping example demonstrates a more uniform estimation of *C_w_* within the fields, with substantially lower uncertainty estimates. This points towards a more adaptive regularization ability of AL-reduced training samples in interpreting real reflectance data.

When it comes to hyperspectral data analysis, dimensionality reduction in the spectral domain should be performed, applying for instance PCA. This step is required to overcome highly correlated information in adjacent bands, often leading to redundant information and noise and hence to sub-optimal retrieval performances [12,31]. The combination of both—DR in spectral and in sampling domains may be key for optimal retrieval results, as demonstrated by our experimental case study. In a future study, the optimal number of components to be applied on hyperspectral data for the retrieval of multiple vegetation traits should be tested.

### 5.3 AL for Hybrid Retrieval Workflows: Ways Forward

AL may be implemented in a number of diverse applications where these specific sampling strategies could support and facilitate the retrieval of multiple vegetation traits from plant/field to satellite scales. With the advent of cloud computing platforms such as the GEE, new opportunities are arising for processing of local-to-global scale satellite data using advanced machine learning algorithms for functional vegetation traits retrieval. For instance the study by Djamai and Fernandes [87] implemented their ALR approach within GEE allowing automated application of multiple solutions in parallel over large datasets based on neural networks. GPR models also have a high potential to become part of these cloud computing environments, but they need to get lighter, which can be efficiently accomplished with implementation of AL methods in the retrieval workflow. Moreover, GPR has the appealing property to provide uncertainty (or confidence) estimates, as opposed to standard neural networks or other statistical methods. This is in particular attractive for mapping applications, allowing to assess the models’ transferability in space and time [5,11]. In the study by Pipia et al. [93], EBD was also evaluated as AL algorithm converging to the highest accuracy with a low number of training samples (for green LAI) and was found suitable for implementation into GEE. As a demonstration case, it enabled mapping the whole Iberian peninsula at 20 m resolution. Accordingly, with support of suitable AL techniques, such a workflow can be realized for mapping multiple vegetation functional traits, e.g., based on Sentinel-2 data, without reaching cloud computing memory limits.

This could also be of interest regarding recently launched and upcoming satellite missions. Our survey revealed that the identified studies mainly exploited multi- and superspectral, or simulated hyperspectral data (see Section 3). However, the expected increase of satellite imaging spectroscopy data (e.g., EnMAP and CHIME) could be a chance to further investigate AL methods for the development of efficient retrieval workflows from time series of these new abundant data streams. Specifically, AL enables generation of an optimized and light training dataset against empirical data for vegetation traits whose retrieval based on RTM simulations is challenging [90].

Emulation can also support optical EO data analysis. These relatively new approaches have not yet been exploited so far in retrieval chains of functional traits mapping and monitoring in combination with AL. For instance, emulators can be used to generate synthetic scenes based on complex RTMs, which are constructed using machine learning regression combined with AL frameworks. Providing realistic scenes of the (terrestrial) Earth’s surface plays a key role in the development of new space instruments specifically designed for vegetation monitoring [113,114]. Moreover, by replacing a computationally expensive RTM with its emulated surrogate, the model inversion process can become extremely fast and hence attractive for processing of large scenes [114].

An appealing application of AL-based methods can be analysis of data gathered during high-throughput field phenotyping experiments [115]: up to now, information content collected at the plant or organ level remains rather under-exploited, in particular regarding the implementation of RTMs [116]. Since exact knowledge of biochemical traits such as nitrogen is critical for plant phenotyping, AL-based hybrid workflows could support more accurate estimations and thus help to optimize fertilizer application optimization in precision agriculture [117].

RTMs are complex, highly nonlinear, and typically hierarchical models. Therefore, shallow ML models may not be able to optimally capture all these complex feature relations [14]. This may be a motivation to explore deeper hierarchical model architectures for hybrid approaches, or for learning the nonlinear relationship between remotely sensed signals and functional vegetation traits. DL approaches are able to extract spatio-temporal features automatically [13]. Hence, unlike single (shallow) ML models, DL models can account for more complex, hierarchical relations and processes, providing efficient solutions that often improve prediction accuracy over shallow models [14]. The study by Wang et al. [46] presented as first the combination of DL with AL for image classification. However, different problems arise within such a framework [45]. For instance, DL algorithms usually require a large amount of labeled training data, whereas in AL scenarios only a small amount of labeled data is available. Nevertheless, a few more studies combined both approaches for diverse applications, concluding that the synergistic usage of DL and AL outperforms the state-of-the-art methods with less label complexity [45]. An attractive option for remote sensing image analysis within regression problems could be the combination of AL with deep Gaussian process (DGPs) regression [14].

Another promising application of AL heuristics could be the exploitation over coupled vegetation-atmosphere RTMs and corresponding simulated training datasets [8,25]. Here, optimized sampling strategies present an efficient solution for operational satellite-based top-of-atmosphere (TOA) retrieval workflows avoiding computationally expensive datasets for training of kernel-based retrieval models. Within future research lines, assimilation of spectral observations from various sensors could also be achieved through hybrid retrieval frameworks (at both top-of-canopy or TOA levels) with the support of AL methods.

## 6 Conclusions and Future Perspectives

In our study, the background of active learning heuristics for three main applications (classification, emulation and regression) was summarized. This was followed by a systematic literature survey about AL within EO data regression analysis for terrestrial targets. Some practical experiments were then used to demonstrate the concept of AL within hybrid workflows for the retrieval of terrestrial vegetation functional traits.

Whereas AL has been abundantly applied for classification problems, its use in the context of regression and emulation, and in particular for the quantification of vegetation functional traits from remote sensing data, is rather underrepresented.

Following the findings of the literature survey and our experimental example, we recommend the implementation of AL heuristics combined with kernel-based machine learning algorithms (such as GPR) in a retrieval workflow due to the following reasons:

- Use of full datasets may include redundant information, which potentially leads to decreasing retrieval accuracy compared to optimized training datasets;
- Efficient reduction of the training database (up to 80% when given 1k samples) results in decreased computational demand, hence increases processing speed for kernel-based algorithms;
- Lighter models established through AL-based sample selection facilitate their storage within software toolboxes;
- AL-based training datasets queried against in situ data are better adapted to real world situations due to the selective behaviour of the techniques;
- GPR trained with AL-reduced datasets resulted in lower retrieval uncertainties as opposed to training with a full data pool.

Reviewed studies as well as our own experiments suggested that EBD methods performed superior to most others in terms of accuracy and processing speed. Yet, followup research and validation of AL strategies over multiple experiments is required to confirm this finding and to further optimize retrieval workflows with active learning.

Moving ahead, the classification community is at the forefront in developing new AL heuristics. They could be adapted for regression problems to establish lighter training datasets and render samples more representative. This ability presents a prerequisite for implementing hybrid algorithms into cloud computing frameworks with instant processing options, opening up new paradigms for remote sensing image analysis.

In summary, active learning holds strong potential for remote sensing regression problems in view of the upcoming huge data availability through satellite imaging spectroscopy. Herein, GPR models trained over RTM-generated training datasets, which are optimized via AL methods, can be the core of next-generation operational hybrid retrieval schemes. Our survey intends to pursue the use of AL strategies for regression problems in the framework of terrestrial Earth observation monitoring.

## Supplementary Material

Appendix

## Figures and Tables

**Table 1:** Studies using AL strategies for regression problems in the context of terrestrial Earth observation data analysis: remote sensors, estimated vegetation traits (abbreviations in Section 3.2), applied machine learning regression algorithms (ML algorithm, abbreviations in Sections 3.2 and 3.3) and active learning strategies (AL method, best performing in **bold**, abbreviations in Section 2.3, Appendixes A.1 and A.2).

References	Sensors	Estimated Traits	ML Algorithms	AL Methods
Verrelst et al. [41]	Sentinel-3 OLCI (simulated)	LAI, *C_ab_*	KRR, GPR	**PAL**, **EQB**, RSAL, **EBD**, ABD, CBD
Upreti et al. [27]	Sentinel-2	LAI, *C_ab_*, Fcover, fAPAR	GPR	**EBD**, ABD, CBD
Verrelst et al. [90]	EnMAP (resampled)	*N_area_*	KRR, VHGPR	**PAL**, EQB, RSAL, **EBD**, ABD, CBD
Upreti et al. [91] Zhou et al. [92]	VEN*μ*S Landsat-8 OLI	Fcover *C_ab_*	GPR GPR	EBD PAL, **EQB**, RSAL, EBD, ABD, CBD
Pipia et al. [93]	Sentinel-2	green LAI	GPR	PAL, EQB, RSAL, **EBD**, ABD, CBD

## Data Availability

Not applicable.
