# Supplementary material for: A Survey of Active Learning for Quantifying Vegetation Traits from Terrestrial Earth Observation Data
Source: Remote Sens (Basel). Author manuscript; Available in PMC 2022 Sep 7. (PMC7613397; doi:10.3390/rs13020287)
Supplement: Appendix [file EMS152662-supplement-Appendix.pdf]

Author Contributions: Conceptualization, K.B. and J.V.; methodology, J.V. and J.P.R.C.; software, J.P.R.C.; validation, J.V. and K.B.; resources, M.W., L.M.; data curation, T.H. and M.W.; writing–original draft preparation, K.B.; writing–review and editing, J.V., L.M., T.H., M.W.; visualization, K.B.; supervision, J.V.; project administration, J.V. and T.H.; funding acquisition, J.V. and T.H. All authors have read and agreed to the published version of the manuscript.

Funding: Jochem Verrelst was funded by the European Research Council (ERC) under the ERC-2017-STG SENTIFLEX project (grant agreement 755617) and Ramón y Cajal Contract (Spanish Ministry of Science, Innovation and Universities). Katja Berger and Matthias Wocher are funded within the EnMAP scientific preparation program under the DLR Space Administration with resources from the German Federal Ministry of Economic Affairs and Energy, grant number 50EE1923. Luca Martino is supported by the Spanish government with the project number PID2019-105032GB-I00.

Institutional Review Board Statement: Not applicable.

Informed Consent Statement: Not applicable.

Data Availability Statement: Not applicable.

Acknowledgments: This publication is also the result of the project implementation: "Scientific support of climate change adaptation in agriculture and mitigation of soil degradation" (ITMS2014+ 313011W580) supported by the Integrated Infrastructure Operational Programme funded by the ERDF. Further, the research was supported by the Action CA17134 SENSECO (Optical synergies for spatiotemporal sensing of scalable ecophysiological traits) funded by COST (European Cooperation in Science and Technology, www.cost.eu). Moreover, we thank the five reviewers for their valuable suggestions.

Conflicts of Interest: The authors declare no conflict of interest.

Appendix A

All active learning criteria methods listed in Appendix A are taken from the study by Verrelst et al. [41].

Appendix A.1. Uncertainty Criteria Methods

- Entropy query-by-bagging

Within entropy query-by-bagging (EQB) methods [34], predictions of k different regressors are ranked according to their entropy:

$$H(x) = -\sum_{i=1}^{k} p(x_i) \log p(x_i), \tag{A1}$$

where $p(x_i)$ is the probability of the sample x being predicted by the regressor i. Those samples for which various regressors give similar values have lower uncertainties, being indicated by smaller or negative entropy values. $H(x)$ is calculated for each sample. Samples that show the greatest entropy are added to the final training dataset.

- Residual regression active learning

According to [82], the residual regression active learning (RSAL) method quantifies the systematic errors generated by a regression algorithm. This is accomplished by training a second model (residual model), which estimates the prediction errors, $e(x) = y - \hat{y}$, where y is the actual observed value, and $\hat{y} = \hat{f}(x)$ is the model prediction given the input x. The algorithm selects the samples that exhibit a high prediction error and adds these to the final training dataset.

Appendix A.2. Diversity Criteria Methods

- Angle-based diversity

The angle-based diversity (ABD) strategy [53] measures the diversity between samples using the cosine angle distance, defined as:

$$\angle(x_u, x_l) = \cos^{-1}\left(\frac{\langle x_u, x_l\rangle}{\|x_u\| \cdot \|x_l\|}\right) \quad \text{(A2)}$$

where $\langle x_u, x_l\rangle$ is the inner product between x_u and x_l. The cosine angle is considered high when samples are far away from each other. Therefore, the learning samples showing largest cosine angles with the training data are added to the final dataset.

- Cluster-based diversity

Cluster-based diversity (CBD) methods [118] first group the data using a clustering algorithm, i.e., k-means. The number of clusters k is set to the number of samples to be added during each iteration of the algorithm. Finally, for each cluster, the nearest sample to the cluster centroid is selected as solution and added to the final dataset.

References

1. OECD. *The Space Economy in Figures: How Space Contributes to the Global Economy*; OECD: Paris, France, 2019.
2. Nock, C.A.; Vogt, R.J.; Beisner, B.E. Functional Traits. In *eLS*; American Cancer Society: Atlanta, GA, USA, 2016; pp. 1–8.
3. Guanter, L.; Kaufmann, H.; Segl, K.; Foerster, S.; Rogass, C.; Chabrillat, S.; Kuester, T.; Hollstein, A.; Rossner, G.; Chlebek, C.; et al. The EnMAP Spaceborne Imaging Spectroscopy Mission for Earth Observation. *Remote Sens.* **2015**, *7*, 8830. [CrossRef]
4. Nieke, J.; Rast, M. Towards the Copernicus Hyperspectral Imaging Mission For The Environment (CHIME). In Proceedings of the IGARSS 2018—2018 IEEE International Geoscience and Remote Sensing Symposium, Valencia, Spain, 22–27 July 2018; pp. 157–159.
5. Verrelst, J.; Camps-Valls, G.; Muñoz Marí, J.; Rivera, J.; Veroustraete, F.; Clevers, J.; Moreno, J. Optical remote sensing and the retrieval of terrestrial vegetation bio-geophysical properties—A review. *ISPRS J. Photogramm. Remote. Sens.* **2015**, *108*, 273–290. [CrossRef]
6. Verrelst, J.; Malenovský, Z.; Van der Tol, C.; Camps-Valls, G.; Gastellu-Etchegorry, J.P.; Lewis, P.; North, P.; Moreno, J. Quantifying Vegetation Biophysical Variables from Imaging Spectroscopy Data: A Review on Retrieval Methods. *Surv. Geophys.* **2019**, *40*, 589–629. [CrossRef]
7. Berger, K.; Verrelst, J.; Féret, J.B.; Wang, Z.; Wocher, M.; Strathmann, M.; Danner, M.; Mauser, W.; Hank, T. Crop nitrogen monitoring: Recent progress and principal developments in the context of imaging spectroscopy missions. *Remote Sens. Environ.* **2020**, *242*, 111758. [CrossRef]
8. Estévez, J.; Vicent, J.; Rivera-Caicedo, J.P.; Morcillo-Pallarés, P.; Vuolo, F.; Sabater, N.; Camps-Valls, G.; Moreno, J.; Verrelst, J. Gaussian processes retrieval of LAI from Sentinel-2 top-of-atmosphere radiance data. *ISPRS J. Photogramm. Remote Sens.* **2020**, *167*, 289–304. [CrossRef]
9. Brede, B.; Verrelst, J.; Gastellu-Etchegorry, J.P.; Clevers, J.G.; Goudzwaard, L.; den Ouden, J.; Verbesselt, J.; Herold, M. Assessment of workflow feature selection on forest LAI prediction with sentinel-2A MSI, landsat 7 ETM+ and Landsat 8 OLI. *Remote Sens.* **2020**, *12*, 915. [CrossRef]
10. Berger, K.; Verrelst, J.; Feret, J.B. Retrieval of aboveground crop nitrogen content with a hybrid machine learning method. *Int. J. Appl. Earth Obs. Geoinf.* **2020**, *92*, 102174. [CrossRef]
11. De Grave, C.; Verrelst, J.; Morcillo-Pallarés, P.; Pipia, L.; Rivera-Caicedo, J.P.; Amin, E.; Belda, S.; Moreno, J. Quantifying vegetation biophysical variables from the Sentinel-3/FLEX tandem mission: Evaluation of the synergy of OLCI and FLORIS data sources. *Remote Sens. Environ.* **2020**, *251*, 112101. [CrossRef]
12. Danner, M.; Berger, K.; Wocher, M.; Mauser, W.; Hank, T. Efficient RTM-based training of machine learning regression algorithms to quantify biophysical & biochemical traits of agricultural crops. *ISPRS J. Photogramm. Remote Sens.* **2020**, under review.
13. Reichstein, M.; Camps-Valls, G.; Stevens, B.; Jung, M.; Denzler, J.; Carvalhais, N.; Prabhat. Deep learning and process understanding for data-driven Earth system science. *Nature* **2019**, *566*, 195–204. [CrossRef]
14. Svendsen, D.; Martino, L.; Camps-Valls, G. Active emulation of computer codes with Gaussian processes - Application to remote sensing. *Pattern Recognit.* **2020**, *100*, 107103. [CrossRef]
15. Féret, J.B.; Berger, K.; de Boissieu, F.; Malenovský, Z. PROSPECT-PRO for estimating content of nitrogen-containing leaf proteins and other carbon-based constituents. *Remote Sens. Environ.* **2021**, *252*, 112173. [CrossRef]
16. Verhoef, W. Light scattering by leaf layers with application to canopy reflectance modeling: The SAIL model. *Remote Sens. Environ.* **1984**, *16*, 125–141. [CrossRef]
17. Verhoef, W.; Bach, H. Coupled soil-leaf-canopy and atmosphere radiative transfer modeling to simulate hyperspectral multi-angular surface reflectance and TOA radiance data. *Remote Sens. Environ.* **2007**, *109*, 166–182. [CrossRef]
18. Jacquemoud, S.; Verhoef, W.; Baret, F.; Bacour, C.; Zarco-Tejada, P.; Asner, G.; François, C.; Ustin, S. PROSPECT + SAIL models: A review of use for vegetation characterization. *Remote Sens. Environ.* **2009**, *113*, S56–S66. [CrossRef]
